# GMP-manufactured CRISPR/Cas9 technology as an advantageous tool to support cancer immunotherapy

**DOI:** 10.1186/s13046-024-02993-1

**Published:** 2024-03-01

**Authors:** M Caforio, S Iacovelli, C Quintarelli, F Locatelli, Valentina Folgiero

**Affiliations:** 1 U.O. Cellular and Genetic Therapy of Hematological Diseases, Bambino Gesù Children’s Hospital, IRCCS, Rome, Italy; 2 U.O Officina Farmaceutica, Good Manufacturing Practice Facility, Bambino Gesù Children’s Hospital, IRCCS, Rome, Italy; 3 Department of Life Sciences and Public Health, Catholic University of the Sacred Heart, Rome, Italy; 4 IRCCS Bambino Gesù Children’s Hospital, Viale San Paolo 15, 00146 Rome, Italy

**Keywords:** GMP procedures, CRISPR/Cas-9, Cancer therapy

## Abstract

**Background:**

CRISPR/Cas9 system to treat human-related diseases has achieved significant results and, even if its potential application in cancer research is improving, the application of this approach in clinical practice is still a nascent technology.

**Main body:**

CRISPR/Cas9 technology is not yet used as a single therapy to treat tumors but it can be combined with traditional treatment strategies to provide personalized gene therapy for patients. The combination with chemotherapy, radiation and immunotherapy has been proven to be a powerful means of screening, identifying, validating and correcting tumor targets. Recently, CRISPR/Cas9 technology and CAR T-cell therapies have been integrated to open novel opportunities for the production of more efficient CAR T-cells for all patients. GMP-compatible equipment and reagents are already available for several clinical-grade systems at present, creating the basis and framework for the accelerated development of novel treatment methods.

**Conclusion:**

Here we will provide a comprehensive collection of the actual GMP-grade CRISPR/Cas9-mediated approaches used to support cancer therapy highlighting how this technology is opening new opportunities for treating tumors.

## Background

The incidence and mortality of cancer still remains the principal health issue worldwide. Despite countless progress, much still needs to be done to improve the outcomes of those patients without a valid therapeutic alternative. The Advanced Therapy Medicinal Products (ATMP) oriented to a precision and individualized treatment for the patients have opened a new era for cancer treatment. In this scenario, the genome editing offers a powerful tool for the development of new strategies for treating cancer.

### Good Manufacturing Practice (GMP) guidelines

ATMPs offer a new powerful opportunity for treating, and in some instances, curing diseases (such as cancer) for which there are often no other available treatments. While this has offered an important new therapeutic tool, it has also raised the need to produce drugs following regulations, modalities, and quality standards that ensure safety for patients. In fact, ATMPs are characterized by a very different modalities, use different cell types and, mostly, for the different manufacturing protocols. In particular, ATMP production is a complex manufacturing process and the procedures are still evolving to meet these unique needs. In this regards GMP [1] are the mandatory guidelines governing ATMPs manufacturing. Noteworthy, GMP compliance is mandatory for all products intended for the market and those used for clinical trials.

These guidelines describe the minimum quality standard that a medicines manufacturer must follow to ensure that products are consistently produced and controlled. These are designed to minimize the risks involved in any pharmaceutical production which cannot be avoided or eliminated even testing the final product [2]. Furthermore it is very important to note that the guidelines do not intend to place any restrains on the development of new concepts of new technologies, rather intend to ensure the quality, safety, efficacy and traceability of the product. In fact, any alternative approaches may be implemented by the manufacturers, the important thing is to demonstrate that the alternative approach can meet the same quality standard. Based on the previous considerations, it is important to make the appropriate assessments of the technologies that are being developed and employed for the ATMPs production, before moving from research scale to clinical or commercial manufacturing. For this reason, it is essential to have a very good process development phase. The main goal of process development is to reach a very robust manufacturing process with high efficiency, cost containment, maintenance of quality and safety standards, and overall risk reduction as additional key objectives. To this end, several preclinical studies have already been developed for ready clinical translation [3–5].

### CRISPR/Cas9 technology mechanism of action

Discovered for the first time in 1987 as a defense mechanism in prokaryotes [6]Clustered Regularly Interspaced Short Palindromic Repeats (CRISPR) has greatly improved the field of precise genome editing. The CRISPR system relies on RNA ‘guides’ that drives the site-specific binding of CRISPR-associated (Cas) proteins for mediating DNA or RNA cleavage [7]. The CRISPR system includes three principal types (I, II and III) and 12 subtypes [8]. The type II relies on a single Cas protein, Cas9, to target a specific sequence of DNA. For this reason, the CRISPR/Cas9 has become the most widely adopted genome editing tool [9]. The requirements for recognizing and cut a specific DNA sequence, once paired with a guide RNA (gRNA), are as follows: 1) a site-specific complementarity between a 20-nucleotides (nt) targeting sequence, called the protospacer, that is a part of the CRISPR RNA (crRNA), which together with the transacting crRNA (tracrRNA) generated a single guide RNA (sgRNA), which recruits the Cas9 nuclease to specific DNA sequences, 2) an NGG protospacer adjacent motif (PAM) sequence located at the 3´ of the targeting crRNA/protospacer sequence. It has been observed that, without the PAM sequence, the Cas9 nuclease cannot cleave the target sequence, also if fully complementary to the sgRNA [10].

Once these two criteria are met, the DNA sequence could be targeted and cut by the Cas9/sgRNA system. The design of a specific sgRNA guide sequence allows the detection of double-strand breaks (DSBs) sites where [11] Cas9 binds and cleaves the target DNA sequences, complementary to the crRNA. DSBs, located at approximately − 3 nucleotides before the PAM sequence, are introduced in the target sequence and then the endogenous DNA DSB repair mechanisms rebuild the breaks. The DNA repair machinery is initiated via two most common pathways: non-homologous end joining (NHEJ), which is the predominant repair pathway in most mammalian cells; the less-frequent homology-directed repair (HDR). The NHEJ frequently results in genomic insertions or deletions (indels) which can introduce frameshift mutations that can result in truncated and/or non-functional proteins. Whereas the HDR uses the donor DNA template to precisely repair DSBs for gene modification [12, 13]. In the genome editing procedure, it is possible to design a DNA template, with high homology to the specific target gene locus, containing the aimed genetic change. The procedure of the genome editing could be very challenging because the efficiency of HDR-mediated gene insertion is significantly lower than NHEJ-mediated INDEL formation [14]. Hence, the editing outcomes are the result of the interaction between these two different repair pathways. Furthermore, the CRISPR/Cas9 system can accurately modify the DNA sequences by generating multiple DSBs at specific sites in the genome and, using multiple guide RNAs, it can achieve a multiple genome editing of the target sequence [9]. Because CRISPR/Cas9 system is more effective and easier to perform compared to the other gene editing technologies, such as zinc-finger nucleases (ZFNs) and transcription activator like effector nucleases (TALENs) [15, 16], it can be advantageously applied in the clinical trials that incorporate gene editing for cancer treatment.

**Fig. 1:**
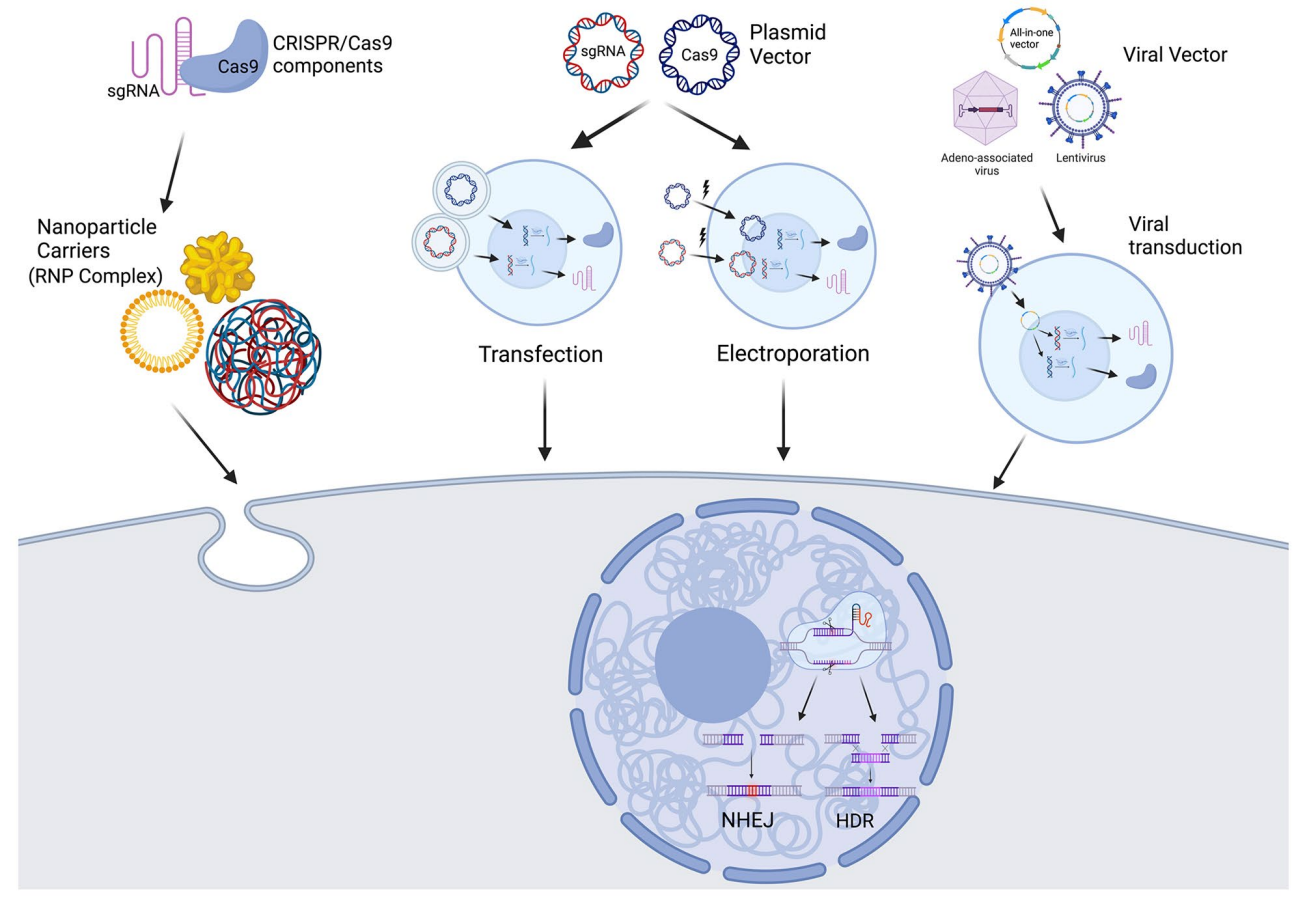
CRISPR/Cas9 mechanism of action. Cas9 and sgRNA vehiculation to edit the nuclear target sequence by nanoparticles when assembled to form RNP complex (left); delivery of the elements as single plasmids of expression through lipo-assisted transfection reagent or by electroporation (center); Viral transduction of Cas9 and sgRNA carrying vector (right). DNA repair machinery (NHEJ, HDR) is activated when the nucleus is reached by the CRISPR/Cas9 system

The CRISPR/Cas9 system is mostly employed in ex vivo strategies to perform gene editing in cells that are then reinfused into the patient. The most commons delivery technologies for gene editing are broadly classified as viral, such as lentivirus, retrovirus, adenovirus and adeno-associated virus, or non-viral vectors, such as electroporation, nanoparticles and cell squeezing (Fig. 1).

### CRISPR/Cas9 clinical applications

The first clinical application of CRISPR/Cas9 system was performed by Lu et al. in 2016, when they carried out in human phase I clinical trial of CRISPR/Cas9 PD-1-edited T cells in patients with advanced non-small-cell lung cancer [17, 18]. Rising from this study, many other clinical trials that use CRISPR/Cas9 in cancer treatment or using gene edited CAR T-cells or Tumor Infiltrating Lymphocytes (TIL) cells have been established (Table 1). Considering this new and powerful opportunity for cancer treatment, it is very important to develop safe and efficient delivery CRISPR/Cas9 system vectors to target the tissues and cells. To be used in clinical trials it is mandatory that these strategies for CRISPR delivery are manufactured following GMP procedures.

**Table 1:** Clinical Trials using CRISPR/Cas9 technology in Cancer Immunotherapy

NCT Number	Study Design	Target Gene	Phases	Cell Type	Tumor Type
NCT04438083	A Safety and Efficacy Study Evaluating CTX130	TRAC; β2M; CD70	Phase 1	CAR T-Cells	Renal Cell Carcinoma
NCT04417764	TACE Combined With PD-1 Knockout Engineered T Cell	PD-1	Phase 1	Engineered T-Cells	Hepatocellular Carcinoma
NCT04244656	A Safety and Efficacy Study Evaluating CTX120	TCR; MHC I	Phase 1	CAR T-Cells	Multiple Myeloma
NCT02793856	PD-1 Knockout Engineered T Cells	PD-1	Phase 1	Engineered T-Cells	Metastatic Non-small Cell Lung Cancer
NCT03081715	PD-1 Knockout Engineered T Cells	PD-1	Completed	Engineered T-Cells	Esophageal Cancer
NCT03545815	CRISPR-Cas9 Mediated PD-1 and TCR Gene-knocked Out Mesothelin-directed CAR-T Cells	PD-1; TCR	Phase 1	CAR T-Cells	Multiple solid tumor
NCT03398967	Safety Study of Universal Dual Specificity CD19 and CD20 or CD22 CAR-T Cell Immunotherapy	TRAC; CD52	Phase 1/ Phase 2	CAR T-Cells	B-cell Acute Lymphoblastic Leukemia
NCT02867332	PD-1 Knockout Engineered T Cells	PD-1	Phase 1	Engineered T-Cells	Renal Cell Carcinoma
NCT05812326	PD-1 Knockout Anti-MUC1 CAR-T Cells	PD-1	Phase 1/ Phase 2	Engineered T-Cells	Breast Cancer
NCT02867345	PD-1 Knockout Engineered T Cells	PD-1	Unknown	Engineered T-Cells	Prostate Cancer
NCT05662904	Genetic Ablation of CD33 in HSC	CD33	Phase 1	Hematopoietic Stem Cells	Acute Myeloid Leukemia
NCT03044743	PD-1 Knockout EBV-CTLs for Advanced Stage Epstein-Barr Virus (EBV) Associated Malignancies	PD-1	Phase 1/ Phase 2	Engineered T-Cells	Gastric Carcinoma; Nasopharyngeal Carcinoma; T cell Lymphoma; Adult Hodgking
NCT03057912	TALEN and CRISPR/Cas9 in the Treatment of HPV-related Cervical Intraepithelial Neoplasia	E6;E7	Phase 1	Engineered T-Cells	Cervical Intraepithelial Neoplasia
NCT05066165	NTLA-5001 in Subjects With Acute Myeloid Leukemia		Phase 1/ Phase 2	CAR T-Cells	Acute Myeloid Leukemia
NCT03747965	PD-1 Gene-knocked Out in Mesothelin-directed CAR-T Cells	PD-1	Phase 1	CAR T-Cells	Mesothelin Positive Multiple Solid Tumors
NCT05643742	A Safety and Efficacy Study Evaluating CTX112	TRAC; β2M; CD70	Phase 1/ Phase 2	CAR T-Cells	B Cell-Malignancies
NCT04502446	A Safety and Efficacy Study Evaluating CTX130	TRAC; β2M; CD70	Phase 1	CAR T-Cells	B Cell-Malignancies
NCT03166878	UCART019 in Patients With Relapsed or Refractory CD19 Tumors	TRAC;CD52	Phase 1/ Phase 2	CAR T-Cells	Leukemia and Lymphoma
NCT05566223	CISH Inactivated TILs in the Treatment of NSCLC	CISH	Phase 1/ Phase 2	Engineered T-Cells	Non small cell lung cancer
NCT05795595	A Safety and Efficacy Study Evaluating CTX131	TRAC; β2M; CD70	Phase 1/ Phase 2	CAR T-Cells	Renal cell carcinoma; Cervical Carcinoma; Pancreatic Adenocarcinoma; Malignant Pleural Mesothelioma
NCT04035434	A Safety and Efficacy Study Evaluating CTX110	TRAC; β2M; CD70;	Phase 1/ Phase 2	CAR T-Cells	B Cell-Malignancies
NCT04426669	CISH depletion using CRISPR/Cas9 in Tumor Infiltrating Lymphocytes	CISH	Phase 1/ Phase 2	Engineered T-Cells	Gastrointestinal Cancer
NCT05037669	Allogeneic CRISPR-edited T Cells Engineered to Express Anti-CD19 Chimeric Antigen Receptor	TCR, HLA-I; HLA-II	Phase 1	CAR T-Cells	Acute Myeloid Leukemia; Chronic Lymphocytic Leukemia; Non Hodgkin Lymphoma
NCT02863913	PD-1 Knockout Engineered T Cells for Muscle-invasive Bladder Cancer	PD-1	Phase 1	Engineered T-Cells	Bladder Cancer

In a very interesting study, Palmer DC et al. [3] developed a clinical scale and GMP-compliant manufacturing process for highly efficient and precise CRISPR/Cas9 CISH knockout (KO) in human T cells and TIL. In several clinical trials the genome editing of the biological component of the study is associated to chemotherapy. Based on the study of Palmer and colleagues, a phase I/II trial has been started for patients with metastatic gastrointestinal epithelial cancer (NCT04426669), in which Cyclophosphamide, Fludarabine and Aldesleukin are administered combined with TIL in which the gene encoding CISH has been inactivated using the CRISPR/Cas9 System. To be administered to the patients the TIL production and the gene editing procedure need to be performed in a GMP grade environment with a quality system that guarantees the final release of genetically modified cells. Another interesting application for CRISPR/Ca 9 system is in the cancer immunotherapy with CAR T-cells. It has been demonstrated that PD-1 deficient CAR T-cells have an improved antitumor activity in vitro [19] while a previous study and clinical trials (NCT02808442 and NCT02746952), performed using TALEN as gene editing system, have showed how disrupting genes encoding T cell receptor (TCR) α and β chains in the infused CAR T-cell product can prevent graft-versus-host disease (GVHD) appears [20]. Based on these results several other clinical trials have been established. In the phase I study for patients with mesothelin positive multiple solid tumors (NCT03545815), a CRISPR/Cas9 mediated gene knock-out of PD-1 and endogenous TCR for CAR T-cells is performed. Following a similar strategy, a phase I trial to assess the safety and feasibility of administering pre-manufactured allogeneic T cells from healthy donors expressing CD19.CAR T-cells lacking expression of HLA class I, HLA class II molecules and endogenous TCR through CRISPR/Cas9 mediated genome-editing of beta-2 microglobulin (β2M), CIITA and T cell receptor alpha chain, respectively (Table 1, NCT05037669).

All of these studies showed the fundamental GMP grade manufacturing role for producing a CRISPR/Cas9 gene edited cell product for clinical trials. In fact several studies are now performed for developing production process for improving clinical scale manufacturing of genetically modified cells for clinical trials [4, 21]. In this review we will provide a comprehensive collection of the actual GMP-grade CRISPR/Cas9-mediated approaches used to support cancer therapy, highlighting how this technology is opening new opportunities for treating tumors.

## Main text

### CRISPR/Cas9 gene-editing of immune check-points

Immunotherapy is a novel approach to fight the growth and invasion of tumor cells by inducing the stimulation of the immune system [22]. It involves cytokine therapy, oncolytic virus therapy, dendritic cell (DC) therapy, cancer vaccine, adoptive cellular immunotherapy (ACT), immune checkpoint blockade, and antibody-drug conjugate (ADC). Additionally, CAR T-cells therapy has demonstrated high efficacy for hematological and recently for solid tumors [23, 24].

Tumor immunity promotes tumor progression by modifying tumor biological features [25], selecting tumor cells adapted to the microenvironment [26] or creating a favorable tumor microenvironment [27]. Among the factors that play an important role in tumor immunity, immune checkpoints molecules such as PD-1 and CTLA4 deserve a special mention. Under physiological conditions, PD-1, expressed on T-cells, binds its physiological ligand, PD-L1, expressed on tumor cells. This interaction may impair the activity of T-cells and prevent further damage induced by cytotoxic effector molecules and autoimmunity.

In recent years immune checkpoint blockade became one of the most important therapeutic options for cancer. Several anti- PD-1/PD-L1 antibodies (Nivolumab, Pembrolizumab, and Atezolizumab) have shown significant advantages in certain malignancies such as melanoma, non-small-cell lung cancer (NSCLC) and urothelial carcinoma, and have been approved by the Food and Drug Administration [28–30]. However, specific side effects remain [31, 32], and the overall survival rate is not significantly improved [33].

CRISPR/Cas9 is an RNA-guided endonuclease, which is widely used as a simple and fast method to modify the DNA of mammalian cells [34]. In primary T-cells, researchers have conducted several studies to test the effectiveness of CRISPR/Cas9 in vitro. Schumann and colleagues introduced prassembled sgRNA and Cas9 endonuclease into human CD4^+^ primary T-cells using electroporation. This delivery resulted in inducing site-specific mutations in CXCR4 and PD-1 genes [35]. Su S. and colleagues conveyed CRISPR/Cas9 system through electroporation in the peripheral CD8^+^ T-cells of cancer patients or healthy individuals. Disruption of PD-1 In T-cells increased immune responses against cancer antigens [36, 37].

The knock-out of PD-1 in T-cell lymphocytes reduces the number of regulatory T-cells (Treg) or impairs Treg activity and recruits more effector cells. In addition, it can modulate the production of cytokines and activate caspases, inhibiting tumor proliferation in vivo and in vitro and improving survival [37–39].

In light of the recent pre-clinical results, CRISPR/Cas9 technology seems to be a good candidate to provide a powerful and effective protocol for editing genes that express checkpoint inhibitors, in particular PD-1, in a wide range of immune cells to block immune checkpoints [33].

Thus, in recent years, numerous clinical trials have been started with the aim to evaluate the potential of gene editing and to translate this knowledge into clinical settings [40].

All therapeutic drugs, including CRISPR/Cas9, during clinical trials must follow GMP procedures in order to minimize steps involved in any pharmaceutical production that cannot be eliminated through final product testing. Since immunotherapy obtained by the CRISPR/Cas9 system has not yet been deeply programmed in clinical practice, many clinical trials are in progress. In fact, in vitro, the combined effect of CRISPR/Cas9 with immunotherapies has been demonstrated such as the improvement of antibody performance [41, 42], the modulation of TME and immune cell activity [43–46] and reprogramming MHC specificity (correcting MHC mismatches) [47].

**Fig. 2:**
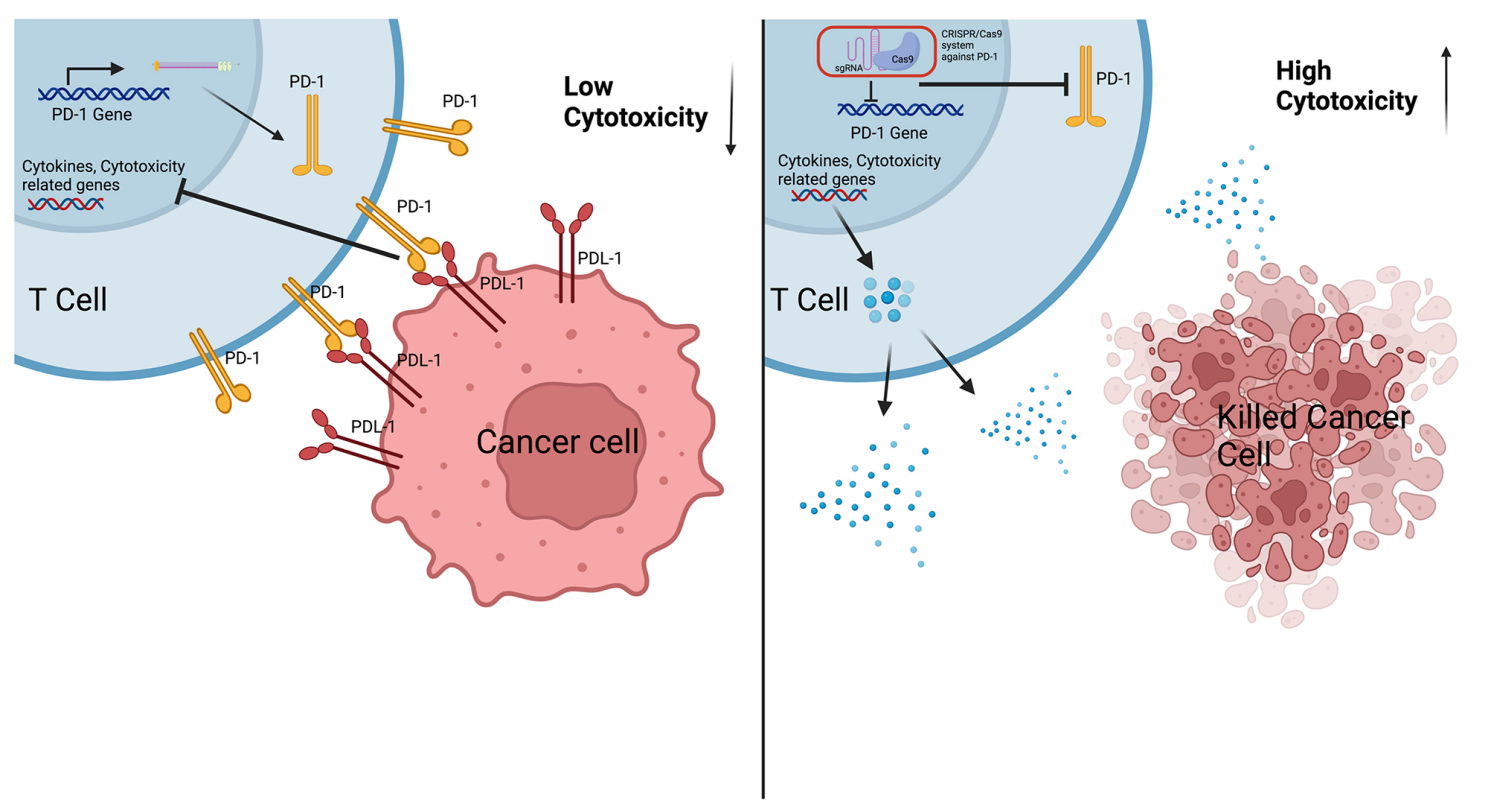
CRISPR/Cas9-mediated editing of T-cell. Interaction between PD-1 receptor transcribed by T-cells and PD-L1 ligand expressed on cancer cells. Activation of PD1/PD-L1 checkpoint inhibits cytokine production and cytotoxic activity of T-cell (left). CRISPR/Cas9-mediated editing of the PD-1 sequence inhibits PD-1 expression allowing extracellular cytokines release and improving T-cell killing activity (right)

But the main focus regards the editing of T-cells. Among the targets of CRISPR/Cas9, PD-1 is the most targeted checkpoint in T-cell. In particular, many clinical trials have focused their attention on the autologous origin T-cell, in which CRISPR/Cas9 system is used to deplete PD-1 (Fig. 2).

For example, in order to evaluate the safety of CRISPR/Cas9 technology, Lu et al. used the CRISPR/Cas9 method to obtain PD-1 depletion in T-cells from patients with non-small-cell lung cancer (NSCLC). The editing of autologous T-cell was followed by ex vivo reinfusion, hypothesizing that may ameliorate T-cell response. PD-1-edited T-cells were modified by co-transfection performing electroporation of Cas9 and sgRNA plasmids. Monitoring of T-cells modifications by next generation sequencing resulted in mutation frequency of off-target events of about 0.05% at 18 candidate sites. The authors conclude that clinical application of CRISPR/Cas9 gene-edited T-cells is generally safe and feasible and that this approach is clinically feasible (NCT02793856) [18].

The knockout of PD-1 performed using the CRISPR/Cas9 system is also used in Epstein-Barr virus cytotoxic lymphocytes (EBV-CTL) cells to treat patients affected by EBV positive advanced stage malignancies. Also in this case, the editing is obtained through CRISPR/Cas9-mediated PD-1 knockout in T-cells of autologous origin. The authors evaluate adverse events after each cycle by Common Terminology Criteria for adverse events as primary endpoint. Tumor and immunological markers are also evaluated as secondary endpoints to monitor the efficacy of the anti-tumor effect (NCT03044743) [48].

Among combination therapies involving PD-1 disruption in T-cells, an important study analyzes the safety and efficacy of a therapeutic vaccine in combination with the depletion of PD-1 carried out by the CRISPR/Cas9 system in the treatment of advanced prostate cancer. The therapeutic vaccine consists in a customized product involving the use of a recombinant fusion protein (PAP-GM-CSF) to stimulate the production of the antigen that would increase the immune system activity to kill tumor cells [49]. The strategy used by the authors was once again the engineering of patient’s T-cells through CRISPR/Cas9 technology to disrupt PD-1 gene. The therapeutic vaccine and PD-1 knockout T cells will be infused back to the patient in 3 times with a 2-week interval and the safety and efficacy effect will be evaluated at the end of the study (NCT03525652).

Another trial investigates the safety and effect of transcatheter arterial chemoembolization (TACE), a minimally invasive therapy that combines local delivery of chemotherapy with a procedure called embolization, in combination with engineered T-cells modified by CRISPR/Cas9 on PD-1 gene in patients with advanced hepatocellular carcinoma. TACE would block the blood supply of the tumor to achieve ischemic, hypoxic and necrotic effects (NCT04417764).

Among immune-checkpoint, the role of Cytokine-inducible SH2 domain-containing protein (CISH)has recently been deeply understood. CISH belongs to the suppressor of cytokine signaling (SOCS) family of negative feedback regulators that have been demonstrated a pivotal role in lymphoid cell function and development. Thus, it is a novel intra-cellular immune checkpoint and an important negative regulator of T-cell able to impair their activity [50].

Tumor Infiltrating Lymphocytes (TIL) have demonstrated efficacy in some malignancies, principally melanoma. Efficacy in most common solid tumors was shown through the selection of cancer neoantigen-specific TIL. Combined therapy with checkpoint inhibitor molecules has also been employed with the aim to increase the efficacy of the therapies with the autologous TILs. Since genetic engineering of T-cells performed by CRISPR/Cas9 that may ameliorate anti-tumor activity is now possible, researchers have improved and optimized a CRISPR/Cas9 based methodology to achieve precise and efficient editing in primary human T-cells without affecting cell function or viability, obtaining the inhibition of undruggable intracellular checkpoint. Thus, researchers are trying to edit the gene encoding this new intracellular checkpoint target, CISH, in TIL obtained from patients with metastatic cancers. Trials that regard the targeting of CISH in TIL through CRISPR/Cas9 involve Metastatic Gastrointestinal Cancers (NCT04426669) and NSCLC (NCT05566223). In these trials the safety and efficacy of genetically modified T-cell selected for anti-tumor activity for solid tumors are evaluated in the setting of novel target that involved checkpoint inhibitor [51].

In the last years scientists have evaluated the possibility to use TALEN and CRISPR/Cas9 to treat human cervical intraepithelial neoplasia induced by Human Papillomavirus (HPV) without invasion. In fact, the infection of HPV is the main causative factor of cervical intraepithelial neoplasia (CIN) and cervical cancer. HPV vaccines strategy allows to target the two most important oncoproteins expressed by HPV16 and HPV18, E6 and E7, which are also constitutively expressed by cancer cells [52, 53]. Numerous strategies have been applied to develop therapeutic vaccines using vectors, peptides/proteins, DNA and genome editing tools. Vector, peptide and protein vaccines are used in particular to treat HPV16 infection, whereas DNA vaccines and the vaccines that use genome editing tools are mostly polyvalent vaccines used for the treatment of both HPV16 and HPV18 and target E6 and E7 genes. The important roles of E6 and E7 playing in HPV-driven carcinogenesis make them attractive targets for therapeutic interventions. Furthermore some experimental studies demonstrated that using TALEN and CRISPR/Cas9 as genome editing tool may induce depletion of E6 and E7 genes, significantly decreasing the expression of E6/E7, inducing cell death and inhibiting cell lines growth [54, 55].

The efficacy and safety of E6/E7 disruption induced by TALEN and CRISPR/Cas9 technology in treating HPV Persistency and HPV-related Cervical Intraepithelial Neoplasia is under evaluation of a specific clinical study (NCT03057912).

NTLA-5001 is an investigational CRISPR/Cas9-engineered T-cell receptor (TCR)-T cell therapy in development for the treatment of all genetic subtypes of acute myeloid leukemia (AML) using a WT1-targeting TCR. This study is conducted to evaluate the safety, tolerability, cellular kinetics (CK), activity, and pharmacodynamics (PD) of NTLA-5001 in participants with AML (NCT05066165).

### CRISPR/Cas9 gene-editing of CAR T-cells

Over the last 30 years, adoptive T-cell transfer has become the major form of cancer immunotherapy, used, predominantly, in hematological malignancies. With this approach, tumor-specific cytotoxic T-cells are infused into patients, upon lympho-depleting chemotherapy [56–58]. The key potential advantage of this treatment strategy is the ability to reach privileged niches where conventional anticancer therapeutics have struggled to penetrate [59]. CAR T-cells usually identify cell surface antigens present in the natural state on the surface of tumor cells without the necessity of peptide processing or HLA expression for recognition [60]. The two most diffused safety-related problems, due to CAR T-cell administration, have been partially overcome. The first, concerning the targeted destruction of normal cells, is resolved through the identification of tumor-specific cell surface molecules to be targeted. The second concern, regarding the possible induction of a cytokine storm associated with anti-tumor response mediated by large numbers of activated T-cells is strongly overcome utilizing suicide genes such as inducible caspase-9 to halt deleterious responses [61, 62]. The innovative principle of CAR T-cells is to couple the potency of a T-cell with the specificity of an antibody to selectively kill target cells. Modifications applied to subsequent generations of CAR T-cells achieved a very efficient product in which inhibitory domains were eliminated and co-stimulatory domains were introduced.

Engineering a patient’s own T-cells to selectively target and eliminate tumor cells has cured patients with untreatable hematological cancers [62, 63] and the manufacturing of CAR T-cells under GMP is a focal point for this therapeutic modality [64, 65]. The main challenges for CAR T-cell therapy concern solid tumors due to the difficulty to identify truly specific tumor antigens as targets, overcoming tumor antigen escape, improving CAR T-cells trafficking, infiltration and expansion at the tumor site as well as persistence and functions in a hostile tumor microenvironment (TME) [66]. Many clinical trials are on going testing CAR T-cells in brain tumors targeting several antigens such as Disialogangloside GD2, to test the promising data obtained at pre-clinical level [67–71].The same target resulted strongly valid in the NCT05573097 clinical trial against high-risk pediatric neuroblastoma showing a sustained anti-tumor effect [72].

Preparation of clinical-grade CAR T-cells for therapy begins with leukopheresis to obtain large numbers of peripheral blood mononuclear cells followed by cryopreservation of these cells. After being thawed, at the manufacturing facility, the cells are activated by CD3/CD28 stimulation for ex-vivo expansion [73].

**Fig. 3:**
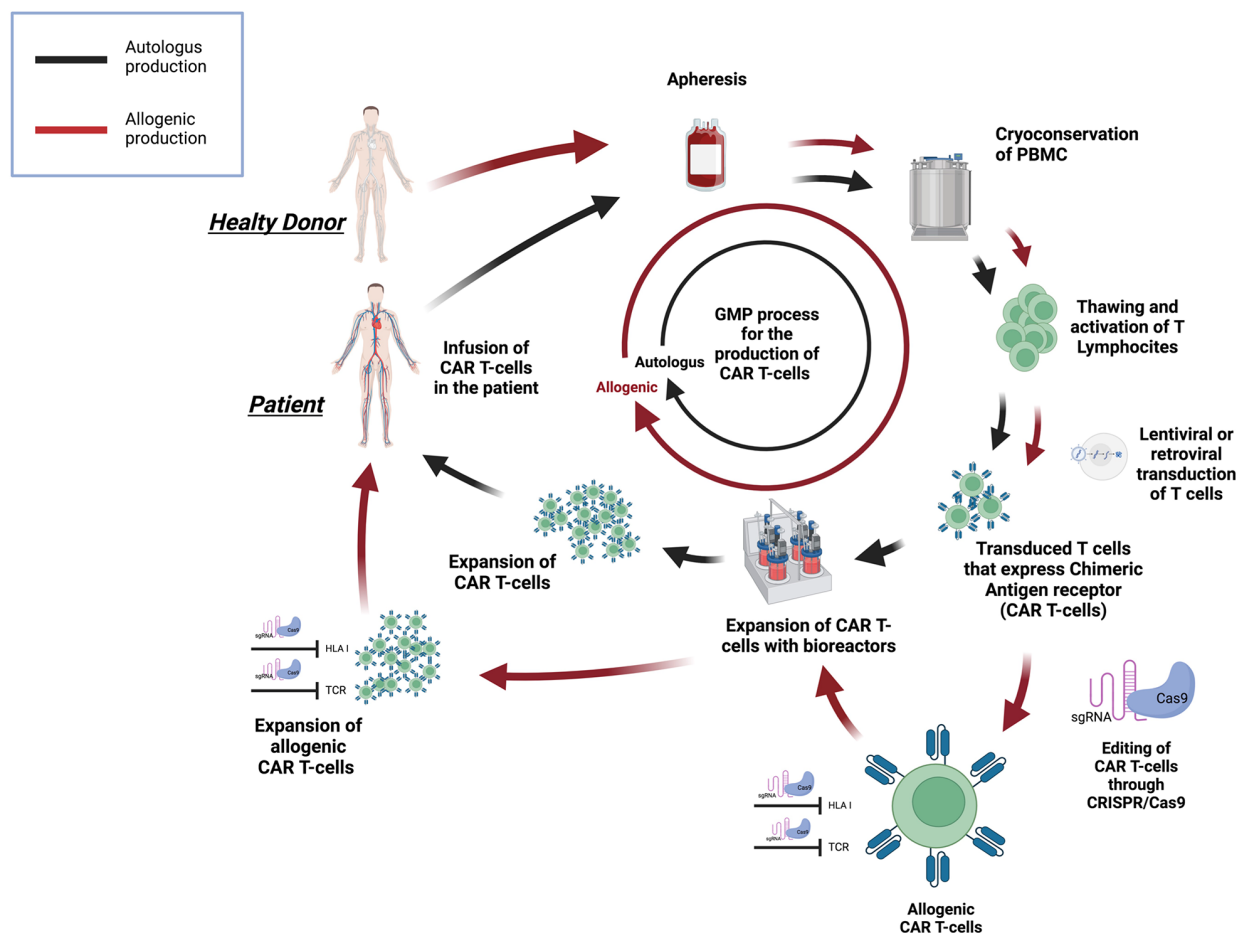
CRISPR/Cas9-mediated editing of CAR T-cell. Production of CAR T cell starting from patient for autologous infusion (black line) including apheresis from peripheral blood and cryo-conservation; thawing is followed by stimulation and lentiviral transduction for generation of CAR T-cells. The expansion is achieved by bioreactors and the requested number of CAR T-cells is ready for patient infusion. The process starting from healthy donor (red line) for allogenic CAR T cell production follows the same procedure until CAR transduction. CRISPR/Cas9 editing of HLA and TCR before CAR T-cells expansion generates universal CAR T-cells that can be infused into the patient

Then, genetic modification of T-cell is carried out through transduction with a self-inactivating lentiviral or retroviral vector encoding the transgene of interest. The transgenic T-cells are expanded through different platform (GE bioreactors, G-Rex bioreactors) until sufficient numbers for treatment are obtained, around 300 million cells. Transduction efficiency is measured by flow cytometry and percent of killing activity is evaluated against tumor cell lines expressing the target antigen [74](Fig. 3).

The use of virus in CAR T-cells production has showed some disadvantages including an increased risk of tumor development resulting from insertional mutagenesis [75].

Despite the success of CAR T-cells in treating hematological malignancies, challenges such as cytokine release syndrome (CRS) [76], T-cell exhaustion, tumor antigen masking [77, 78] and durability and risk of GVHD remain [79].

For this reason, recently, CRISPR/Cas9 technology has been integrated with CAR-T cell-based treatment to open novel opportunities for the production of more efficient CAR-T cells for all patients [80–82].

Non-viral gene-editing systems can be delivered to primary T cells using electroporation, liposome or nanoparticle transfection methods [83]. The best tool that meets the three crucial criteria which are lack of immunogenicity, compatibility with GMP grade reagents and feasibility on a clinical scale is electroporation [84]. A non-viral protocol to generate gene-specific integrated T-cells was developed in 2021 by Jiqin Zhang et al. An anti-CD19 CAR sequence containing 4-1BB and CD3z was constructed and electroporated into T cells. Through this procedure, cell expansion was not impaired and cell viability was high. In addition, electroporation increased the ratio of CD8^+^ to CD^+^4 T cells when compared to lentiviral transduction. Since blocking the PD1-PD-L1 axis has been demonstrate to improve CAR T-cells killing activity, the authors integrated an anti-CD19 sequence into the PD1 gene obtaining a robust clearance of tumor cells. Safety and efficacy assay, followed by GMP-procedures adaptation, was performed to carry out a phase I clinical trial (NCT04213469) in B-NHL patients and in relapsed/refractory B-cell malignancies (NCT04637763, CB010). Data obtained by the trial revealed that the development of non-viral gene-specific targeted CAR T-cells by CRISPR/Cas9 showed high efficiency against the tumor through a simplified manufacturing procedure with reduced preparation time and expenses.

Although autologous T-cells have shown promising results in many cases, there are many patients that cannot be treated in this way or for lymphocyte repertoire depletion due to myeloablative therapies, or for intrinsic defect of autologous T-cells. These limitations can be overcome by developing universal genetically engineered CAR T-cells derived from allogenic donor T-cells where TCR and HLA-I are silenced. CRISPR/Cas-9 can be used to knock-out β2M of donor CAR T-cells, a component that forms heterodimers with HLA-I and is requested for HLA-I surface expression, and to silence TCRα subunit constant (TRAC) or TCRβ gene (TCRB) to eliminate the recognition of alloantigen of the recipient. Although at a preclinical level, this study demonstrates that CRISPR/Cas9-mediated multiplex gene editing is applicable and a relay promising strategy [85]. Indeed, a recent phase I clinical trial (CARBON) shows how CTX-110, an anti CD19 CAR T-cell in which MHC I complex has been eliminated by CRISPR/Cas9 editing of TCRA and β2M administrated in patient with relapsed/refractory Diffuse Large B-cell Lymphoma (DLBCL) resulted highly efficient (NCT04035434).

Antigen-escape-mediated relapse is another limitation CAR T therapy and the use of multiantigen targeting could allow the optimization of the response. Yongxian Hu et al. proposed combined approach using universal CD19/CD22 dual targeting CAR T-cells in which TRAC and CD52 gene region is disrupted by using CRISPR/Cas9 technology. The phase I clinical trial (NCT04227015) in adult patients with relapsed/refractory B-cell acute lymphoblastic leukemia showed a safety profile and prominent anti-leukemia activity, especially for patients that were ineligible for autologous CAR T-cells administration [86]. Recently gene editing supported also allogenic “off-the-shelf” CAR T-cells targeting B-Cell Maturation Antigen (BCMA) in multiple myeloma (CTX-120) using CRISPR/Cas9 system to eliminate TCR and MHC class I, coupled with specific insertion of the CAR at the TRAC locus [87]. Results from animal models showed complete tumor regression and phase I study is ongoing in patients with refractory or relapsed multiple myeloma (NCT04244656). A valid study was performed in clear renal cell carcinoma through the development of allogenic CRISPR/Cas9-engineered CAR T-cells. It was designed to insert an anti-CD70 CAR cassette into the TRAC locus to disrupt TRAC, b2M and CD70 CTX-130). The results from phase I trial (NCT04438083) showed safety and encouraging antitumor activity [88].

## Conclusions

The latest advancements in GMP procedures are allowing an efficient improviement of personalized medicine. In particular immunotherapy is strongly taking advantage of clinical manufacturing platforms to cure patients who are refractory to previous therapy or who relapse upon a first period of remission. CRISPR/Cas9 technology has become the most widely used gene-editing tool in cancer immunotherapy favoring differentiation and persistence of genetically modified T-cells. The discovery of cancer-selected markers remains one of the principle obstacles while the use of allogenic CRISPR/Cas9-modified CAR T-cells is overcoming the difficulties to treat relapsing cancer cells showing an antigen different from that expressed at the onset of the disease. CAR T-cells generated against multiple tumor targets are preventing relapsing events. The manufacturing processes still comprised procedures performed manually even if supported by semi-automated manner, which result in product variability and very high cost. The development of a more controlled and cost-effective manufacturing process remains the pivotal aim to ensure CAR T-cells therapy for all patients.

## Abbreviations

CRISPR: Clustered Regularly Interspaced Short Palindromic Repeats
Cas9: CRISPR Associated Protein 9
CAR: Chimeric Antigen Receptor
GMP: Good Manufacturing Practice
ATMP: Advanced Therapy Medicinal Products
gRNA: Guide RNA
NT: Nucleotide
crRNA: CRISPR RNA
tracrRNA: Transacting crRNA
sgRNA: Single Guide RNA
PAM: Protospacer Adjacent Motif
DSBs: Double-Strand Breaks
NHEJ: Non-Homologous End Joining
HDR: Homology-Directed Repair
INDEL: Insertions or Deletions
ZFNs: Zinc-Finger Nucleases
TALENs: Transcription Activator Like Effector Nucleases
PD-1: Program Cell Death 1
TIL: Tumor Infiltrating Lymphocytes
CISH: Cytokine-Inducible SH2 Domain-Containing Protein
KO: Knock Out
TCR: T-Cell Receptor
GVHD: Graft Versus Host Disease
HLA: Human Leukocyte Antigen
β2M: Beta-2 Microglobulin
CIITA: Class II Trans Activator
DC: Dendritic Cell
ACT: Adoptive Cellular Immunotherapy
ADC: Antibody-Drug Conjugate
CTLA4: Cytotoxic T-Lymphocyte Antigen 4
PD-L1: Program Cell Death Ligand-1
NSCLC: Non Small Cell Lung Cancer
CXCR4: Chemokine Receptor Type 4
TReg: T-Regulatory-Cell
TME: Tumor Microenvironment
MHC: Major Histocompatibility Complex
EBV-CTL: Epstein-Barr Virus Cytotoxic Lymphocytes
PAP-GM-CSF: Prostatic Acid Phosphatase-Granulocyte Macrophage-Colony Stimulating Factor
TACE: Transcatheter Arterial Chemoembolization
SOCS: Suppressor of Cytokine Signaling
HPV: Human Papillomavirus
CIN: Cervical Intraepithelial Neoplasia
AML: Acute Myeloid Leukemia
WT-1: Wilms Tumor-Suppressor Gene-1
CK: Cellular kinetics
PD: Pharmacodynamics
CRS: Cytokine Release Syndrome
B-NHL: B-Lymphoma Non Hodgking
TRAC: TCRα Subunit Constant
TCRB: T Cell Receptorβ
DLBCL: Diffuse Large B-cell Lymphoma
BCMA: b-Cell Maturation Antigen
